# Pilot trial of a type I - polarized autologous dendritic cell vaccine incorporating tumor blood vessel antigen-derived peptides in patients with metastatic breast cancer

**DOI:** 10.1186/2051-1426-3-S1-P3

**Published:** 2015-08-14

**Authors:** Joseph Baar, Walter J Storkus, James Finke, Lisa Butterfield, Hillard Lazarus, Jane Reese, Katharine Downes, Thomas Budd, Adam Brufsky, Pingfu Fu

**Affiliations:** 1 Seidman Cancer Center, Cleveland, Ohio, USA; 2 University of Pittsburgh Cancer Institute, Pittsburgh, Pennsylvania, USA; 3 Taussig Cancer Center, Cleveland, Ohio, USA

## 

Cancer vaccines based on tumor-associated antigens are rarely curative in advanced cancer. This limitation relates to the heterogeneity of cancer due to defects in antigen presentation and altered immunophenotypes. Therefore, another method to promote anti-tumor immunity is to prime T cells against tumor-associated stromal cells. We have reported [1] that IL-12 gene therapy of established HLA-A2neg B16 melanomas in HLA-A2+ transgenic mice resulted in CD8+ T cell-mediated immunity against the host HLA-A2+ stromal cells within the tumor microenvironment (TME). We have also shown [2] that vaccines based on a subset of tumor blood vessel antigen (TBVA)-derived peptides (DLK1, EphA2, HBB, NRP1, PDGFRβ, RGS5 or TEM1) prevented HLA-A2neg MC38 tumor establishment and promoted the regression of tumors in HLA-A2+ mice by CD8+ T cell targeting of HLA-A2+ pericytes and vascular endothelial cells in the TME. Based on this pre-clinical data, we propose to undertake a Susan G. Komen -funded (IIR13261822) clinical trial of chemo-immunotherapy using the immunomodulatory drug gemcitabine with a dendritic cell vaccine pulsed with six HLA-A2-presented TBVA-derived peptides (DLK1310-318, EphA2883-891, HBB31-39, NRP1433-441, RGS55-13 and TEM1691-700) in 30 HLA-A2+ patients with metastatic breast cancer. The specific aims of this study are to determine vaccine-induced generation of TBVA-Tc1 immunity and clinical response.
